# Hepatitis E 3ra Genotype Infection in People Living With HIV in Spain

**DOI:** 10.3389/fmicb.2020.564486

**Published:** 2020-09-11

**Authors:** Antonio Rivero-Juarez, Mario Frias, Pedro Lopez-Lopez, Juan Berenguer, Federico García, Juan Macias, Begoña Alcaraz, Angeles Castro-Iglesias, Javier Caballero-Gomez, Antonio Rivero

**Affiliations:** ^1^Instituto Maimonides de Investigación Biomédica de Córdoba, Hospital Universitario Reina Sofía de Córdoba, Universidad de Córdoba, Córdoba, Spain; ^2^Hospital General Universitario Gregorio Marañón, Instituto de Investigación Sanitaria Gregorio Marañón, Madrid, Spain; ^3^Hospital Universitario San Cecilio, Instituto de Investigación Biosantaria Ibs, Granada, Spain; ^4^Hospital Nuestra Señora de Valme, Seville, Spain; ^5^Hospital General Universitario Santa Lucía, Cartagena, Spain; ^6^Complejo Hospitalario Universitario a Coruña, A Coruña, Spain; ^7^University of Córdoba - Agrifood Excellence International Campus, Córdoba, Spain

**Keywords:** hepatitis E, HIV, survey, genotype 3ra, seroprevalence

## Abstract

**Background:**

The objective of our study was to assess the prevalence and incidence of HEV in people living with HIV (PLWH) in a Spanish national cohort.

**Methods:**

Retrospective longitudinal study including PLWH recruited in the cohort of adult HIV-infected patients of the AIDS Research Network in follow-up at 28 Spanish hospitals with available serum samples in 2014 and 2015. All samples were tested for HEV IgG, IgM, and RNA. Samples with detectable HEV viral loads were genotyped. Prevalence and incidence of HEV infection were calculated.

**Results:**

The study sample comprised 845 PLWH. At baseline, 101 patients were positive for HEV IgG antibodies (11.9%), none had HEV IgM antibodies, and 2 presented detectable HEV RNA (0.23%). Forty-two seroconverted for IgG, supposing a cumulative incidence of 5.7%. One subject was positive for IgM (0.13%), and 2 showed detectable HEV RNA (0.27%). One case was infected by the emergent HEV genotype 3ra.

**Conclusion:**

Our study identifies one case of HEV 3ra genotype infection, the main host of which is rabbit, showing a potential zoonotic role of this emerging genotype in Spain.

## Introduction

Hepatitis E virus (HEV) is an emerging infectious disease worldwide. In Europe, the majority of cases are related to local infections, represented by HEV genotype 3 (Aspinall et al., 2017). Although HEV infection by this autochthonous genotype usually presents as subclinical or self-limiting acute hepatitis, there are several related complications that substantially worsen the prognosis of the infection (Faber et al., 2018). HEV genotype 3 has tropism for the peripheral and central nervous system, producing neurological injury, including serious manifestations such as Guillen-Barré syndrome (Dalton et al., 2017). On the other hand, immunosuppressed patients infected by HEV genotype 3 could develop chronic infection (Kamar et al., 2008). Cases of chronic HEV infection have been described in transplant recipients, leukemia patients treated with rituximab, patients on therapy with anti-tumoral necrosis factor drugs, and subjects infected by HIV with a low CD4 + cell count (Rivero-Juárez et al., 2020). Finally, in the context of underlying liver disease, HEV infection can trigger liver failure and may require liver transplantation (Frias et al., 2018). Consequently, there are subsets of patients in whom HEV could have a serious outcome. Among the population in which HEV infections may have a worse prognosis, persons living with HIV (PLWH) represent a high-sensitivity population because of the high rate of advanced liver fibrosis due to common hepatotropic virus coinfection and the underlying immunosuppression (Rivero-Juarez et al., 2019).

In Europe, the main HEV genotype is 3 (Abravanel et al., 2017; Oeser et al., 2019; Suin et al., 2019). Nevertheless, in recent years, an important number of new viral strains have emerged that encompass a growing caseload in countries such as Switzerland, France, Belgium and Spain (Abravanel et al., 2017; Caballero-Gómez et al., 2019; Oeser et al., 2019; Suin et al., 2019). The emergence of these new strains has an important clinical implication because the introduction of new viral strains could have a negative impact in terms of an increased number of symptomatic and worst prognosis cases (Oeser et al., 2019). In addition, recently, there has been awareness about the zoonotic behavior of several HEV viral strains, the main host of which are lagomorphs and rodents (Abravanel et al., 2017; Sridhar et al., 2018; Suin et al., 2019). These strains have been described in a high proportion of patients with chronic hepatitis, suggesting that those patients with underlying immunosuppression could be more suitable for infection by these emerging viruses (Sridhar et al., 2018; Sahli et al., 2019).

For these reasons, our study assessed the prevalence and incidence of HEV, as well as the prevalence of chronic HEV infection, in a large national cohort of PLWH, describing the HEV viral strains.

## Materials and Methods

### Study Population

The cohort of adult HIV-infected patients of the AIDS Research Network (CoRIS) is an open, prospective, multicentre cohort of adult subjects with confirmed HIV infection who are naïve to antiretroviral therapy (ART) at cohort entry and who are recruited to HIV care units of the Spanish Public Health System (Caro-Murillo et al., 2007), which constitutes the standard place of treatment for the great majority of persons in Spain. CoRIS was launched in 2004. Each center recruits into the cohort all subjects seen for the first time at the center who meet the following criteria: confirmed HIV diagnosis, and naïve to ART. Written informed consent is obtained from all patients. Demographic, clinical, laboratory, microbiological and treatment information is recorded. The cohort is linked to a centralized BioBank, where patients’ blood samples are processed and cryopreserved immediately after reception and then stored (García-Merino et al., 2009). Participating centers are encouraged to obtain an initial blood sample at entry in the cohort, preferentially before starting ART, and follow-up samples preferentially annually, or at least biannually thereafter. The BioBank has obtained the UNE-EN-ISO 9001:2008 Systems of Quality Management Requirements. For the proposed study, we included patients recruited in the cohort at follow-up in 28 hospitals belonging to 15 Spanish provinces with available serum samples in the centralized BioBank in 2014 (baseline) and 2015.

### HEV Serological and Molecular Determination

First, all patients were tested for HEV IgG and IgM antibodies. Serum samples were tested for anti-HEV IgG and IgM (Wantai HEV-IgM ELISA^®^; Beijing Wantai Biological Pharmacy Enterprise^©^ LTD, Beijing, China). ELISA tests were carried out in duplicate in accordance with the instructions provided by the manufacturer, using the automated ELISA Triturus System (Grifols S. A, Barcelona, Spain). The cut-off value for positive samples was > 1.1, following the manufacturer’s instructions. Patients who were positive for HEV anti-IgG antibody at baseline (prevalence) were not evaluated for IgG antibodies in the follow-up (incidence).

Second, all patients were tested for HEV RNA using RT-qPCR. RNA was extracted from 400 μL of serum pools of 4 patients using the commercial QIAamp MinElute Virus Spin Kit (QIAgen. Hilden, Germany) and an automated procedure (QIAcube. QIAgen, Hilden, Germany). The purified RNA was eluted in a total elution volume of 30 μL. As a positive extraction control, 50 μL of the reconstituted WHO Standard HEV strain (supplied by the Paul-Ehrlich-Institute [code 6329/10]) was diluted in 350 μL of Ambion^®^ DEPC-treated water (Thermo Fisher Scientifics. Waltham, MA, United States). RT-qPCR was performed on all pooled samples using the CFX Connect (Bio-Rad, Hercules, California), using primer and probe designed and validated by our group and described recently (Caballero-Gómez et al., 2020). These primers and probes were obtained by aligning all whole-genome sequences of the *Orthohepevirida*e A species available in GenBank. The procedure was validated using the WHO international reference panel for HEV RNA genotypes for nucleic acid amplification technique (ıNAT)ı-based assays (including genotypes 1a, 1e, 3b, 3c, 3e, 3f, 3ra, 4c, 4g, and 2a) supplied by the Paul-Ehrlich-Institut (code 8578/13). For the reaction, the QIAgen One-Step PCR Kit (QIAgen, Hilden, Germany) was used using 25 μL of RNA template. An external (in-run) standard curve (using 18 dilutions) was used to calculate the HEV viral load using the WHO Standard HEV strain (code 6329/10) with a unitage of 250,000 International Units/mL. Positive pools were individually evaluated by the same RT-qPCR procedure. The sensitivity of PCR in individual samples was 21 IU/mL, and in 4 samples-pools it was set at 350 IU/mL.

Samples with detectable HEV viral loads were genotyped according to the protocol described by *HEVnet*. For phylogenetic analysis, nested RT-PCR was performed, targeting the ORF2 region (structural proteins), using primers HEV_5920S (5′-CAAGGHTGGCGYTCKGTTGAGAC-3′) and HEV_6425A (5′-CAAGGHTGGCGYTCKGTTGAGAC-3′) in the first round and HEV_5930S (5′-GYTCKGTTGAGACCWCBGGBGT-3′) and HEV_6334A (5′-TTMACWGTRGCTCGCCATTGGC-3′) in the second round. The second amplification product of 467 bp was sequenced using the BigDye Terminator Cycle Sequencing Ready Reaction Kit on an ABI PRISM 3100 Genetic Analyzer (Applied Biosystems, Foster City, CA, United States). SnapGene software (Version 3.1; GSL Bio-tech, snapgene.com) was used for sequence analysis. The consensus sequence was obtained using SeqMan Software SeqMan NGen^®^ Version 12.0 (DNASTAR. Madison, WI). Subtype assignment and phylogenetic analyses were performed using the *HEVnet* genotyping tool^1^ (Mulder et al., 2019), and confirmed by BLAST. Sequence alignments were generated by the MAFFT online service: multiple sequence alignment, interactive sequence choice and visualization. Phylogenetic trees were constructed using the maximum likelihood method using the proposed HEV genotype/subtype standard reference (Smith et al., 2016). Reference sequences of genotype 4 were included as an outgroup to root the tree. The final tree was obtained with MEGA Software (Version 6) using the bootstrap method (bootstrapped with 1000 replicates).

### Statistical Analysis

Three outcome variables were established: (i) positivity for IgG antibodies against HEV, (ii) positivity for IgM antibodies against HEV, and (iii) detection of HEV RNA. These variables were evaluated at baseline. The prevalence of IgG, IgM, and HEV RNA was calculated. For those patients who were negative for all outcome variables, three outcome variables were evaluated at the end of the follow-up: (i) seroconversion for IgG antibodies against HEV, (ii) seroconversion for IgM antibodies against HEV, and (iii) detection of HEV RNA. The incidence rate of HEV was calculated. The accumulated incidence was expressed as a percentage. Continuous variables were expressed as the median and quartiles (Q1–Q3). Student’s *t*-test, the Welch test or the Mann-Whitney *U*-test was used to compare two independent variables, and a Kruskal-Wallis test, one-way ANOVA or Welch test was used to compare more than two independent variables. The most appropriate test was chosen based on a normal distribution (using the Shapiro-Wilk test) or equality of variances (using the Levene test). Categorical variables were expressed as the number of cases (percentage) compared using the χ^2^-test or Fisher’s exact test. Significance was determined as a two-tailed *p*-value of less than 0.05. Bivariate analysis comparing the prevalence and incidence rate between groups was carried out to identify variables related to the different outcome variables. All analyses were carried out using the SPSS statistical software package version 18.0 (IBM Corporation, Somers, NY, United States), GraphPad Prism version 7 (Mac OS X version; GraphPad Software; San Diego, California, United States) and Winpepi software version 11.36 (Brixton Health).

### Ethics

This study was designed and performed according to the Helsinki Declaration. The local and national Clinical Trial and Ethical Committee approved the study protocol.

## Results

### Study Population and Baseline Characteristics

A total of 845 individuals were included in the study. Seven hundred and fifty-one (88.9%) were male and had a median age of 36.9 years (30.7–45.2 years). Regarding HIV-related variables, 616 (72.9%) were men who had sex with men (MSM), and 80 (9.4%) had prior AIDS-defining conditions, with a median CD4 + cell count of 574 cells/mL (413–787 cells/mL), and 410 (48.5%) with detectable HIV viral load. Because patients are included in the cohort if they are naïve to antiretroviral therapy, the population presents a high proportion of patients with a detectable viral load.

### Prevalence and Incidence of HEV Infection

One hundred and one patients showed positivity of IgG against HEV (11.9%; 95% CI: 9.9–14.3%). There were no variables associated with IgG positivity in the univariate and multivariate analyses (Table 1). Among the previously described risk factors for HEV in HIV-infected patients, neither men who had sex with men (MSM) nor CD4 + cell counts were associated with a higher rate of HEV seroprevalence. Any of the patients included in the study were positive for IgM anti-HEV. Two patients showed detectable HEV viral load (0.23%; 95% CI: 0.0–0.9%). Both patients were male and were negative for both IgG and IgM antibodies.

**TABLE 1:** Univariate and multivariate analysis for IgG anti-HEV at baseline.

**Variable**	**IgG anti-HEV positive (*N* = 101)**	**IgG anti-HEV negative (*N* = 744)**	**Univariate *p*-value**	**Adjusted OR (95% CI)**	**Multivariate *p*-value**
**Age (year), mean (SD)**	36.2 (19.8)	36.3 (14.3)	0.926	0.997 (0.98–1.01)	0.652
**Gender, n (%)**					
*Male*	86 (11.5)	665 (88.5)	0.236	1	
*Female*	15 (16)	79 (84)		0.905 (0.37–2.21)	0.828
**Country of origin, n (%)**					
*Western Europe*	84 (12.3)	599 (87.7)	0.917		
*Eastern Europe*	1 (5.6)	17 (94.4)			
*North-Central America*	0	6 (100)			
*Caribbean*	3 (10)	27 (90)			
*South America*	10 (11.8)	75 (88.2)			
*Asia*	0	4 (100)			
*Africa*	3 (16.7)	15 (83.3)			
**HIV acquisition route, n (%)**					
*Heterosexual*	24 (14.6)	140 (85.4)	0.643		
*MSM*	67 (10.9)	549 (89.1)			
*PWID*	5 (15.2)	28 (84.8)			
*Other*	1 (20)	4 (80)			
*Unknown*	4 (14.8)	23 (85.2)			
**HIV acquisition route, n (%)^a^**					
*MSM*	67 (10.9)	549 (89.1)	0.134	1	
*No MSM*	34 (14.9)	172 (85.1)		0.738 (0.38–1.42)	0.363
**AIDS defining criteria, n (%)**					
*No*	88 (11.5)	677 (88.5)	0.207	1	
*Yes*	13 (16.2)	67 (83.8)		2.02 (0.99–4.13)	0.052
**Time since HIV diagnosis, mean (SD)**	3.67 (4.5)	3.58 (3.8)	0.823		
**CD4 + cell count, mean (SD)^b^**	586 (307)	611 (292)	0.474		
**CD4 + cell count, n (%)^b^**					
<200	5 (9.6)	47 (90.4)	0.622		
200–349	11 (16.7)	55 (83.3)			
350–500	19 (12.8)	130 (87.2)			
>500	50 (11.4)	389 (88.6)			
**CD4 + cell count, n (%)^b^**					
<200	5 (9.6)	47 (90.4)	0.824	1	
≥200	80 (12.2)	574 (87.8)		0.61 (0.22–1.67)	0.343

*IgG, Immunoglobulin G; HEV, hepatitis E virus; OR, odds ratio; SD, standard deviation; n, number of patients; HIV, human immunodeficiency virus; MSM, men who have sex with men; PWID, people who inject drugs. ^*a*^Twenty-seven patients with unknown acquisition routes were excluded from the analysis. ^*b*^One-hundred and thirty nine patients did not have CD4 + cell values.*

Of the 744 patients negative for IgG antibodies at baseline, 733 (98.5%) had available serum samples in the follow-up. Forty-two patients showed seroconversion for IgG antibodies after 1 year of follow-up, supposing a cumulative incidence of 5.7% (95% CI: 4.3–7.7%). Similar to the prevalence analysis, by univariate and multivariate analysis, all factors were not associated with HEV seroconversion during the follow-up (Table 2). One patient showed both positive IgG and IgM antibodies but did not have detectable HEV RNA. HEV RNA was detected in two patients, but they were negative for IgG and IgM antibodies.

**TABLE 2:** Univariate and multivariate analysis for IgG anti-HEV seroconversion during follow-up.

**Variable**	**IgG anti-HEV positive (*N* = 42)**	**IgG anti-HEV negative (*N* = 691)**	**Univariate *p*-value**	**Adjusted OR (95% CI)**	**Multivariate *p*-value**
**Age (year), mean (SD)**	36 (21)	36 (14)	0.279	0.99 (0.96–1.02)	0.735
**Gender, n (%)**					
*Male*	37 (5.6)	619 (94.4)	0.794	1	
*Female*	5 (6.5)	72 (93.5)		0.99 (0.21–4.67)	0.998
**Country of origin, n (%)**					
*Western Europe*	34 (5.8)	557 (94.2)	0.463		
*Eastern Europe*	0	17 (100)			
*North-Central America*	0	2 (100)			
*Caribbean*	3 (11.5)	23 (88.5)			
*South America*	4 (5.5)	69 (94.5)			
*Asia*	1 (25)	3 (75)			
*Africa*	0	15 (100)			
**HIV acquisition route, n (%)**					
*Heterosexual*	9 (6.5)	129 (93.5)	0.518		
*MSM*	30 (5.5)	511 (94.5)			
*PWID*	1 (3.6)	27 (96.4)			
*Other*	1 (25)	3 (75)			
*Unknown*	1 (4.5)	21 (95.5)			
**HIV acquisition route, n (%)^a^**					
*MSM*	30 (5.5)	511 (94.5)	0.652	1	0.205
*No MSM*	11 (6.5)	159 (93.5)		0.44 (0.12–1.56)	
**AIDS defining criteria, n (%)**					
*No*	38 (5.7)	628 (94.3)	0.788	1	
*Yes*	4 (6)	63 (94)		2.11 (0.56–7.92)	0.267
**Time since HIV diagnosis, mean (SD)**	5.4 (6.9)	3.5 (3.8)	0.081	1.05 (0.96–1.16)	0.245
**CD4 + cell count, mean (SD)^b^**	630 (238)	672 (280)	0.662		
**CD4 + cell count, n (%)^b^**					
<200	1 (7.1)	13 (92.9)	0.909		
*200*–*349*	1 (3.4)	28 (96.6)			
350–500	6 (6.5)	86 (93.5)			
>500	19 (5.2)	344 (94.8)			
**CD4 + cell count, n (%) ^b^**					
<200	1 (7.1)	13 (92.9)	0.547		
≥200	26 (5.4)	458 (94.6)			

*IgG, Immunoglobulin G; HEV, hepatitis E virus; OR, odds ratio; SD, standard deviation; n, number of patients; HIV, human immunodeficiency virus; MSM, men who have sex with men; PWID, people who inject drugs. ^*a*^Sixteen patients with unknown acquisition routes were excluded from the analysis. ^*b*^Two-hundred and fifty-one patients did not have CD4 + cell values.*

### Phylogenetic Analysis

**FIGURE 1:**
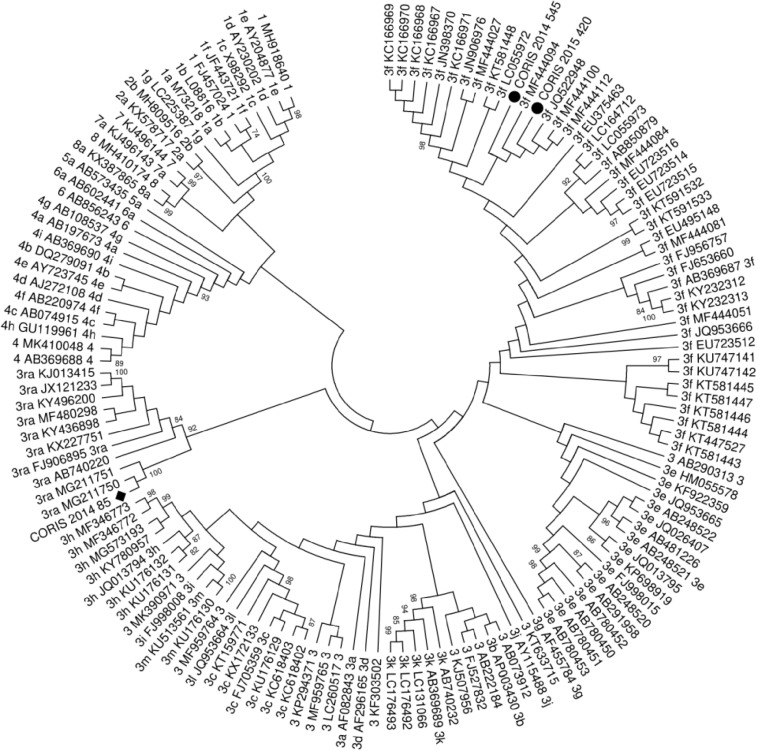
Molecular phylogenetic analysis by Maximum Likelihood method. The evolutionary history was inferred by using the Maximum likelihood method based on the Tamura-Nei model. The bootstrap consensus tree inferred from 1000 replicates is taken to represent the evolutionary history of the taxa analyzed. Branches corresponding to partitions reproduced in less than 50% bootstrap replicates are collapsed. Initial tree(s) for the heuristic search were obtained automatically by applying the Neighbor-Joining and BioNJ algorithms to a matrix of pairwise distances estimated using the Maximum Composite Likelihood (MCL) approach and then selecting the topology with superior log likelihood value. The analysis involved 120 nucleotide sequences. The codon positions included were 1st + 2nd + 3rd + Non-coding. All positions containing gaps and missing data were eliminated. Novel subtypes sequences proposed by *HEVnet* are referred in the tree as p.

Of the 4 patients with detectable viral load in the study, three were successfully genotyped. In two cases, viral strains were consistent with genotype 3f (Genbank accession numbers MN914126 and MN914127). Interestingly, in the other patient, the isolated viral strain was consistent with genotype 3ra (Genbank accession number MN537838) (Table 3). Phylogenetic analysis is shown in Figure 1. No patient had a detectable HEV viral load in the follow-up, indicating spontaneous viral clearance.

**TABLE 3:** Characteristics of patients with detectable HEV RNA.

**ID**	**City**	**Country of origin**	**Year of sampling**	**Gender**	**Age**	**HEV viral load (IU/mL)**	**HEV genotype**
545	Madrid	Germany	2014	Male	43	10,376	3f
85	Tarragona	Spain	2014	Male	52	217,024	3ra
420	Madrid	Spain	2015	Male	43	24,387	3f
466	Murcia	Nigeria	2015	Female	36	3,197	Ungenotyped

*ID, identification number; HEV, hepatitis E virus; IU/mL, International Unit per milliliter.*

## Discussion

The prevalence of HEV among PLWH strongly depends on the country of origin and the immunoassays employed in the study. In this sense, in high endemic areas, such Africa or Asia, the reported seroprevalence is higher than 40%; in addition, in other regions, such as Europe, these data vary between 29 and 10% (World Health Organization [WHO], 2014). Our study found a Spanish seroprevalence of HEV infection of 11.9% and a cumulative incidence of 5.9% in 1 year. These results suggest widespread circulation of HEV among PLWH in Spain.

The main route of transmission of the HEV genotype 3 is the consumption of raw or undercooked meat, including game species and pork (Aspinall et al., 2017; Faber et al., 2018). In two large case series reported in France and Switzerland, the major subtype belonged to clade 3efg and the emerging subtype 3s, respectively (Abravanel et al., 2017; Sahli et al., 2019). In a large temporal series in Belgium, the majority of patients were infected by genotype 3f (Suin et al., 2019). Interestingly, these studies found several cases of HEV infection due to the viral genotype 3ra, whose main host are rabbits. Our study identified one case of HEV infection by genotype 3ra, confirming the zoonotic role of this genotype and the first description of a case of its type in Spain. It should be highlighted that the viral strain identified in our study shows a high similarity with two viral strains isolated in France (MG211750 and MG211751; Figure 1), suggesting the widespread of this strain in Europe. Remarkably, none of the cases found in France and Switzerland documented the consumption of rabbit meat or were in contact with them (Abravanel et al., 2017; Sahli et al., 2019). We cannot determine the source of infection because of the retrospective character of the analysis of the study. Further investigations are needed to evaluate other possible routes of infection of this genotype in addition to consumption or contact with rabbits. Remarkably, in these series, the rate of chronic infection among patients infected with genotype 3ra is higher than those observed in other subtypes. Presently, several cases of chronic HEV infection have been reported in PLWH (Rivero-Juarez et al., 2019). In our study, none of the patients with detectable HEV viral loads developed chronic infections, including those infected with emergence genotype 3ra. Therefore, chronic HEV infection seems to be a rare event in PLWH.

On the other hand, there are several variables related to HIV infection that could be associated with a higher risk for HEV infection in this population. In this sense, the association between the CD4 + cell count and the seroprevalence of HEV is controversial. There are studies that found no differences in the rate of HEV IgG antibodies and the CD4 + cell count (Pineda et al., 2014; Rivero-Juarez et al., 2015; Zeng et al., 2017). Other studies found that this variable could influence a higher or lower rate of infection (Kenfak-Foguena et al., 2011; Zhou et al., 2018). Our study did not find any association between the CD4 + cell count, using different cut-offs, and the presence of IgG antibody, either in the baseline or during the follow-up. In the same way, it is controversial whether MSM constituted a risk population for HEV infection. Studies conducted in Europe report a higher HEV seroprevalence in MSM than in non-MSM populations (Payne et al., 2013; Lanini et al., 2015). In our study, MSM showed a similar HEV seroprevalence and seroincidence to non-MSM. This finding is consistent with a recent study conducted in Asia, where MSM did not show a higher risk for HEV infection in comparison with non-MSMs (Lin et al., 2019), unlike other enteric viruses, such as hepatitis A.

Several limitations should be noted. First, despite the high number of hospitals involved in the present study, there is a lack of patients from different regions. Therefore, our study lacks the power to identify differences in prevalence between regions. Finally, due to the anonymous character of the samples, additional patient data (such epidemiological variables related to risk for HEV acquisition) cannot be evaluated.

## Conclusion

In conclusion, our study found that HEV infection in PLWH from Spain is frequent, showing a relatively high annual incidence. Despite the number of acute infections identified in our study, none of the cases involved chronic infection. We identified one case of infection by the HEV genotype 3ra, the main host of which is the rabbit, confirming the zoonotic role of this emerging genotype.

## Data Availability Statement

The raw data supporting the conclusions of this article will be made available by the authors, without undue reservation to, any qualified researcher.

## Ethics Statement

The studies involving human participants were reviewed and approved by the Comité de Ética de la Investigación de Córdoba. Portal de Ética de la Investigación Biomédica de Andalucia. The patients/participants provided their written informed consent to participate in this study.

## Author Contributions

AR-J and AR: concept and design. JB, FG, JM, BA, AC-I, and AR: collection of samples. MF, PL-L, and JC-G: EIA analysis. MF, PL-L, JC-G, and AR-J: RNA extraction and HEV-PCR. PL-L and AR-J: sequencing and phylogenetic analysis. AR-J, AR, and MF: draft the manuscript. All authors: critical revision of the manuscript. Funding: AR-J, AR, and MF.

## Conflict of Interest

The authors declare that the research was conducted in the absence of any commercial or financial relationships that could be construed as a potential conflict of interest.

## Footnotes

1 https://www.rivm.nl/mpf/typingtool/hev/
